# Activation of Different Signals Identified with Glia Cells Contribute to the Progression of Hyperalgesia

**DOI:** 10.1007/s10571-012-9881-8

**Published:** 2012-10-23

**Authors:** Satoru Yamamoto, Yusuke Kishishita, Mitsuhiro Yoshida, Daisuke Miura, Hidenori Suzuki, Kozo Ishikawa, Hirofumi Miyazaki, Junzo Nojima, Misa Yamamoto, Toshizo Ishikawa

**Affiliations:** 1 Division of Neurosciences, Graduate School of Medicine, Yamaguchi University, Yamaguchi, Japan; 2 Department of Physical Therapy, YIC Rehabilitation College, Yamaguchi, Japan; 3 Division of Dental Anesthesiology, Department of Physical Function, Kyusyu Dental College, Kitakyusyu, Japan; 4 Division of Laboratory, Yamaguchi University Hospital, Yamaguchi, Japan; 5 Department of Orthopedic Surgery, Graduate School of Medicine, Yamaguchi University, Yamaguchi, Japan; 6 Department of Organ Anatomy, Graduate School of Medicine, Yamaguchi University, Yamaguchi, Japan; 7 Division of Laboratory Sciences, Graduate School of Medicine, Yamaguchi University, Yamaguchi, Japan

**Keywords:** BDNF, Hyperalgesia, JNK, Neuroglia interaction, p38-MAPK

## Abstract

Hyperalgesia results from a decreased pain threshold, often subsequent to peripheral tissue damage. Recent reports revealed several promising mechanisms of hyperalgesia, but many issues remain unclear. The glial activation accompanying inflammation of neurotransmission in the spinal cord might be related to the initiation and maintenance of hyperalgesia. The present study investigated the pharmacological pain-modifying effects of mitogen-associated protein kinase (MAPK)-related inhibitors identified with glia cells over time during inflammatory pain. A model of inflammatory pain was produced by injecting mustard oil (MO) into the hind paws of rats. Following MO injection, the changes in paws flinching as the early onset of pain and paw withdrawal latency (PWL) in response to thermal stimulation were measured as delayed-onset hyperalgesia. Before and after the MO injection, one of the inhibitors, a p38-MAPK (SB), nuclear factor (NF)-κB (PDTC), BDNF-trk-B (K252a), or JNK-1 (SP), was administered and flinching and PWL were measured. In the SB, PDTC, and k252a groups, early flinching following MO injection was moderately suppressed. Hyperalgesia was significantly suppressed in the left–right difference of PWL in animals receiving SB, k252a, or PDTC pre-treatment. In animals receiving post-treatment, the suppressive effects were most potent in the SP group. The present results revealed that microglial activation resulting from the release of the phosphatase p38-MAPK, the transcription factor NF-κB, and BDNF contributes to the early stage of inflammatory pain. Astrocyte activation accompanying JNK activation contributes to subsequent hyperalgesia. Activation of different signals identified with glia cells is thought to contribute to the progression of hyperalgesia, which represents an applicable finding for the treatment of hyperalgesia.

## Introduction

Recent studies have suggested that persistent pain is a reflection of neural plasticity that may reflect excessive neurotransmission related to abnormal intracellular signaling and dysfunction of inhibitory neurons in the spinal and supraspinal cord (Woolf and Salter 2000; Zhuo 2007). Intracellular phosphates and members of the mitogen-associated protein kinase (MAPK) family that can modulate glia activity (Svensson et al. 2003; Hua et al. 2005; Ji and Suter 2007) and brain-derived neurotrophic factor (BDNF) derived from neurons or glia (Ji and Suter 2007; Zhou et al. 2008) are thought to contribute to the process of pain facilitation following tissue damage. The MAPKs family represents a group of molecules that plays a critical role in intracellular signaling and gene expression. Inhibition of intracellular signaling may be effective for reduction of pain facilitation (Svensson et al. 2003; Zhuo 2007). Thus, activation of MAPKs is thought to have a key role in signaling after central sensitisation is evoked physically or pharmacologically (Merighi et al. 2008). The major members of this family are extracellular signal-regulated kinase (ERK), p38-MAPK, and c-jun N-terminal kinase (JNK). It is well known that once activated by phosphorylation, MAPK facilitates exaggerated intra-cellular signaling followed by nuclear process ERK, cAMP response element-binding protein (CREB), and nuclear factor-κB (NF-κB). Characterizing changes over time in the phosphatase-related activity associated with these abnormal pain states would help elucidation of the mechanisms underlying neuroglial interactions in the pain pathways.

Spinal glial cells and related activators are thought to contribute to the onset and maintenance of pathological pain, but the specific roles remain unclear. Several investigations on the contribution of spinal microglia and astrocytes over time using ERK phosphatase (p-ERK), a key enzyme in intracellular signaling (Zhuang et al. 2005), and the profile of astrocytic S100β over time (Tanga et al. 2006) represent the only reported forays into this largely uncharted territory.

Thus, we aimed to increase understanding based on the pain-modifying effects of intrathecally administered MAPK-related inhibitors in a rat inflammatory pain model.

### Experimental Procedures

All studies were approved by the ethics committee for animal studies at Yamaguchi University and were carried out according to the Guidelines for Animal Experimentation and the Law (No. 105) and Notification (No. 6) of the Japanese Government.

### Animals and Intrathecal Catheter Placement

Male Sprague–Dawley rats (260–350 g; Kyudo Co., Ltd., Saga, Japan) were used in this study. Under a 12-h light (07:00–19:00)/dark cycle and a room temperature of 23 ± 2 °C, rats were provided ad libitum access to food and water. Three days before the experiments, rats were anesthetized with halothane (2–3 %) and immobilized at the head, then a 9-cm polyethylene tube (PE-10) for drug delivery was inserted into the cisterna magna, with the tip placed so as to coincide with the lumbar spine (L_1–2_) according to the methods of Yaksh and Rudy (1976).

### Surgical Operation for a Model of Inflammatory Pain

According to the methods of Ishikawa et al. (1999), rats were again anesthetized with halothane (2–3 %), and a 30-G needle was used to inject 50 μL of 20 % allyl isothiocyanate (mustard oil: MO:, 016-01463; Wako Pure Chemical Industries, Osaka, Japan) diluted with mineral oil (150138; MP Biomedical, LLC, Tokyo, Japan) into the left hind paw. Following MO paw injection, the animals were transferred to a restraining cage (Bollman Cage; Natsume Seisakusho, Tokyo, Japan). With the animals at rest, spontaneous flinching behavior was counted for 8 h and pain thresholds as thermal hyperalgesia were measured at 24 h after MO paw injection.

### Study Paradigm

Rats were assigned at random to a sham group that received anesthesia only and saline, an untreated group (UT group) that received MO injections and saline, and groups that received the drugs stated below. The drugs were administered via spinal catheter before MO injection (pre-treatment) or 4 h after injection (post-treatment). The sham group and UT group received only pre-treatment injections and were used for the comparison with the effects of the other drugs.

1. Sham group: inject 50 μL saline into the left hind paw (No MO paw injection) (*n* = 3)
2. UT group: inject saline 10 μL 60 min before MO injection (*n* = 5)
3. Drug treatments:
  The rats received the following drugs before MO injection (pre-treatment) and 4 h after MO injection (post-treatment).
  1. SB group: 5 μg/10 μL of p38-MAPK inhibitor (SB203580, Calbiochem, Darmstadt, Germany), SB was administered 60 min before MO injection (pre-treatment; *n* = 5, post-treatment; *n* = 3).
  2. PDTC group: 100 μg/10 μL of NF-κB inhibitor (ammonium pyrolidine dithio-carbamate; PDTC) (Sigma-Aldrich Co., Tokyo, Japan). PDTC was administered 60 min before MO injection (pre-treatment; *n* = 3, post-treatment; *n* = 3).
  3. K252a group: 50 μg/10 μL of Trk-B inhibitor (K252a; LKT Laboratories Inc., Saint Paul, USA). K252a was administered 20 min before MO injection (pre-treatment; *n* = 5, post-treatment; *n* = 3).
  4. SP group: 25 μg/10 μL of JNK-1 inhibitor (SP600125; Calbiochem, Darmstadt, Germany). SP was administered 60 min before MO injection (pre-treatment: *n* = 3, post-treatment: *n* = 3).

## Measurements

### Flinching Behavior as Spontaneous Pain

Pain response occurring immediately after MO injection was determined by observing and measuring flinching, a polysynaptic pain response characterized by skeletal muscle contraction in the hind limb on the affected side. Rats were placed in Bollman cages and restrained to allow free movement of the hind limbs. Flinching for 5 min was observed and measured at several measurement intervals before MO injection and during the 8 h after injection. The summation of flinching for 8-h period (Σ flinching/8 h) was calculated to determine the flinching data over time.

### Measurement of Paw Withdrawal Latency (PWL) in Response to Thermal Stimuli

**Fig. 1:**
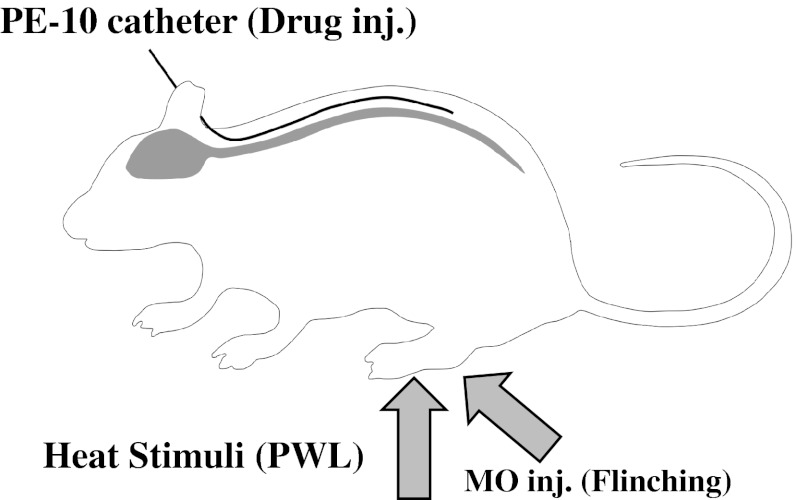
Schematic drawing of experimental design. Rats were intrathecally implanted with a PE-10 catheter for drug administration. Following mustard oil (MO) paw injection, spontaneous pain behavior (flinching/5 min) was measured for 8 h and after that the paw withdrawal latency (PWL, s) against thermal stimuli at 24 h was measured as hyperalgesia using plantar testing

Thermal hyperalgesia was based on paw withdrawal latency (PWL in s) using Hargreaves’ plantar test apparatus (LMS 7370S, 7371, UGO BASILE, Italy). In brief, the rat was placed on a Plexiglas floor and stabilized for 15 min. Constant infrared thermal stimulation was given alternately to the bottom of the hind limbs and the time required for moving the lower limb in response (PWL in s) was measured. PWL in both hind paws was measured five times alternately with an interval of at least 5 min and averaged after rejection of the maximum and minimum values. PWL was measured before and over the 24-h period after MO injection (Fig. 1).

### Immunohistochemistry

At the early stage (1–4 h) or late stage (24 h) after MO paw injection, the animals were anesthetized with halothane (2–3 %) and perfused with saline followed by 4 % paraformaldehyde through the right atrium. The spinal cord was removed and fixed with 10 % formalin on the next day. Sections of the lumber enlargement from the L_3–5_ level were prepared at 10-μm thickness and subjected to immunohistochemistry. The avidin–biotin complex (ABC) method was used to assess changes in the number of activated cells for astrocyte or microglia using anti-glial fibrillary acidic protein (GFAP) rabbit antibody (1:100, Code No. Z0334, Dako Cytomation, Denmark) and anti-Iba1 rabbit antibody (1:1000, Code No. 019-19741, Wako, Kyoto, Japan). After staining with the ABC method, sections were visualized with DAB and positive cells were colored brown in the dorsal horn of spinal cord on each side and were counted at 200×–400× magnification. Histochemical assessments including structural regions according to the Watson and Paxinos rat atlas fourth edition were performed in a double-blind manner and the total numbers of cells counted in three neighboring sections were averaged.

### Statistical Analysis

All data in this study are expressed as the mean ± standard error of the mean. The flinching was evaluated by plotting a time course following MO injection, then calculating the sum of flinching up to 8 h after MO injection. The left–right difference in PWL was calculated up to 24 h after MO injection. The resulting data were analyzed using two-way analysis of variance (two-way ANOVA) and followed by multiple comparisons among averages for flinching (PWL), and cell counts were assessed using the Tukey–Kramer method when significant. Values of *p* < 0.05 were considered as statistically significant.

## Results

### Flinching Response After MO Paw Injection

**Fig. 2:**
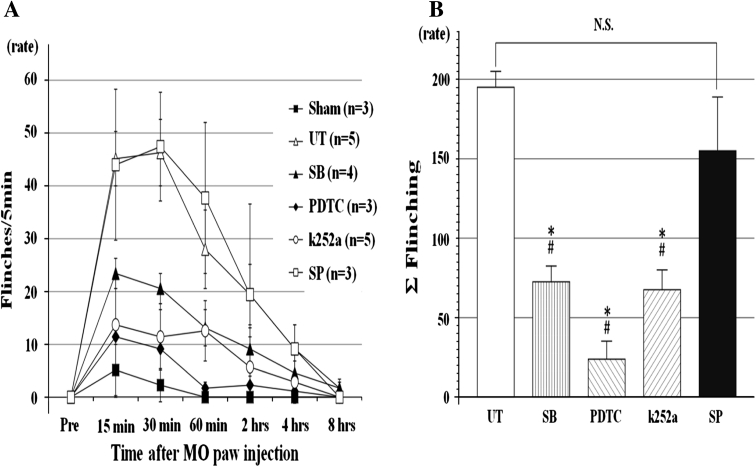
The effects of drugs on flinches induced by MO paw injections in rats. **a** In the untreated group, the peak of flinching was seen at 30–40 min and then *open rectangle* it decreased gradually until 8 h. In the pre-treated SB, PDTC, and k252a group, flinching behavior was restrained moderately, but in the pre-treated SP group, the* curve* was similar to the untreated group. Values are shown as the mean ± SEM.** b** Summation of flinches for 8 h. The flinching behavior was significantly reduced in pre-treated SB, PDTC, and k252a groups, but not in the pre-treated SP group. Values are shown as the mean ± SEM. *p < 0.05 vs. UT; #*p* < 0.05 vs. SP

Figure 2 shows the time course for flinching behavior following MO injection in each group. Saline was injected subcutaneously in the sham group, but only very transiently induced flinching that occurred during measurement. In the UT group, the rats showed a gradual increase in flinching following MO injection, peaking at an average rate of 45/5 min after 30–40 min. Flinching behavior had disappeared almost completely by 8 h after injection. The Σ flinching was significantly reduced by 37 % in the SB group, 13 % in the PDTC group, and 34 % in the k252a group when compared with the UT group. In comparison, Σ flinching in the SP group did not differ significantly from that in the UT group.

### Thermal Hyperalgesia at Late Stage Following MO Paw Injection

**Fig. 3:**
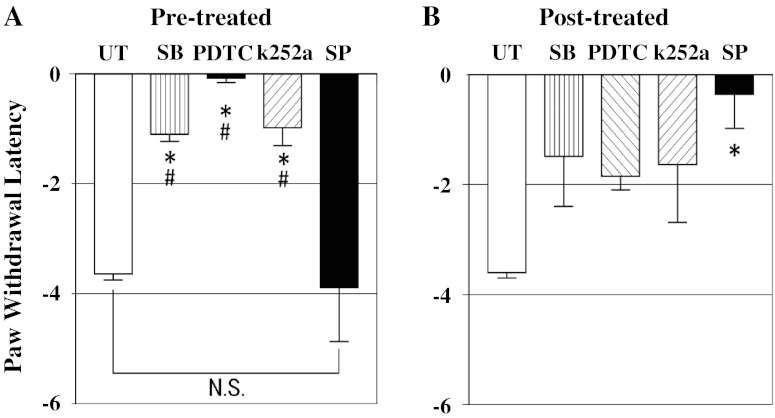
Changes in paw withdrawal latency (PWL) to thermal stimuli at 24 h after MO paw injection. **a** In the pre-treated SB, PDTC, and k252a groups, the decreased PWL in untreated group was significantly reduced, but not in the pre-treated SP group. **b** In the post-treated SP group, the decreased PWL in the untreated group was significantly reduced, but not in the other drug groups. Values are shown as the mean ± SEM. *p < 0.05 vs. UT; #*p* < 0.05 vs. SP

Figure 3 shows changes in PWL in response to thermal stimuli in the different groups 24 h after MO injection. In the UT group, the mean PWL was markedly reduced to −3.62 s. The pre-treatment by each drug showed a significant reduction to this decrease in PWL. The mean PWL was −1.05 s in the SB group, −0.05 s in the PDTC group, and −0.94 s in the k252a group (Fig. 3a). But the mean PWL in the SP group was not significantly different from that in the UT group. When drugs were administered as post-treatment, only the PWL in the SP group was significantly suppressed, comparable to that in the UT group at −0.33 s (Fig. 3b).

### Glial Activations in the Spinal Cord After MO Injection

**Fig. 4:**
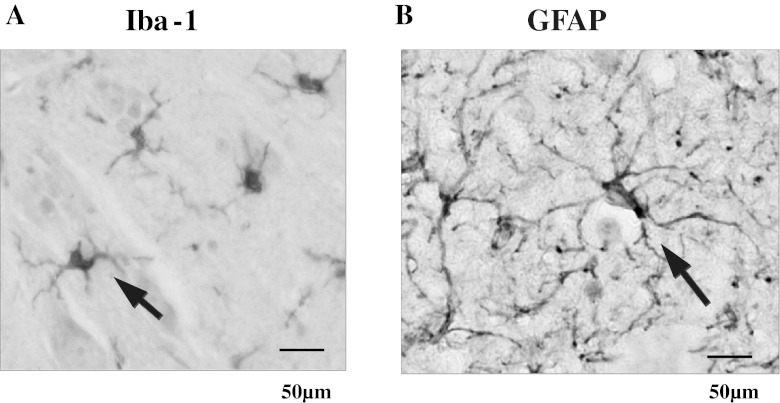
Representative microphotograms for microglia cells (**a**) and astrocytes (**b**) stained by each antibody. *Arrows* indicate positive cells against antibody

**Fig. 5:**
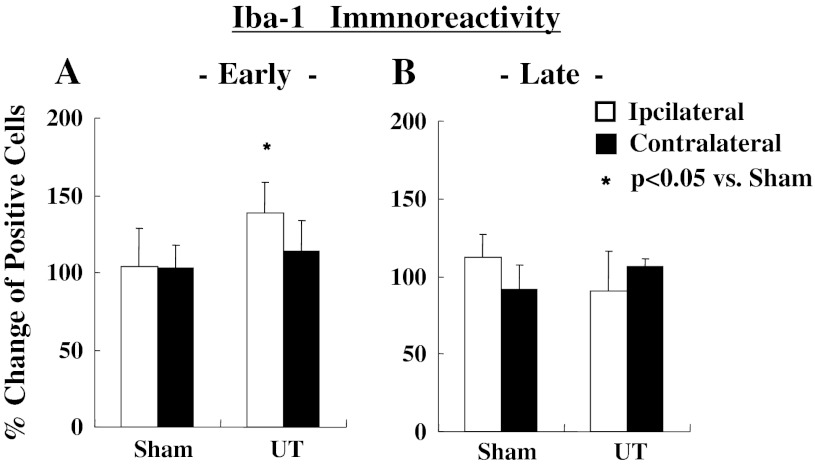
Changes in immunoreactivity in staining of microglia (Iba-1) at 1 and 8 h after MO injection. Activated microglia (Rexed I–II) increased on 1 h (**a**), but there were no changes at 8 h (**b**). Values are shown as the mean ± SEM. *p < 0.05 vs. Sham

**Fig. 6:**
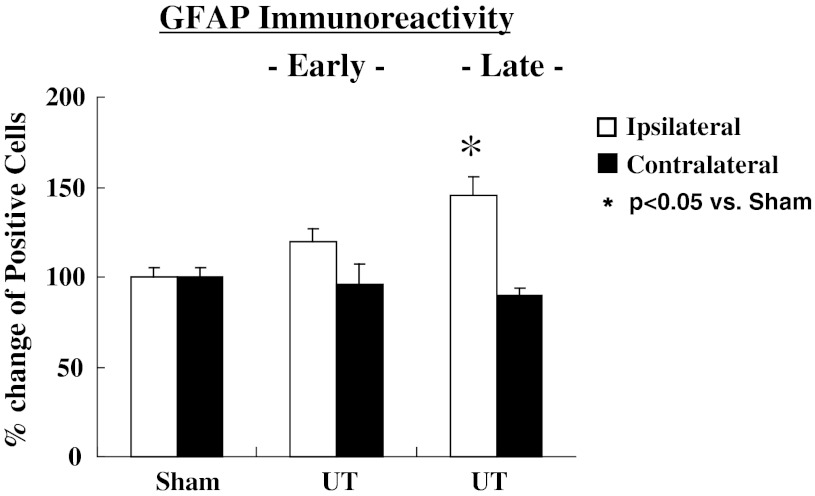
Changes in immunoreactivity in staining of astrocytes (GFAP) at 8 and 24 h after MO injection. MO had no influence on activated astrocyte immunoreactivity at 8 h, but increased at 24 h. Values are shown as the mean ± SEM. *p < 0.05 vs. Sham

The representative photograms for Iba-1 (microglia) and GFAP (astrocytes) are shown in Fig. 4. The activated microglia cells based on expression of Iba-1 on the ipsi-lateral side of the spinal cord increased significantly at early (*p* < 0.05), but had no change at the late stage as shown in Fig. 5. In contrast, a delayed increase (late stage) in activated astrocytes expressing GFAP on the ipsi-lateral side of the spinal cord (*p* < 0.05) is shown in Fig. 6.

## Discussion

This study was conducted to characterize the contribution of neuroglia-related signaling in the spinal cord to the progression of inflammatory pain with phosphatases and BDNF used as markers. In the early stage of hyperalgesia, microglial activation was related to p38-MAPK, and transcription factor NF-κB activity and BDNF were associated with this contribution. Moreover, astrocytic activation related to JNK activation contributed to delayed hyperalgesia. Activation of different signals identified with glia cells is, therefore, thought to contribute to the progression of hyperalgesia over time.

### Characteristics of the Inflammatory Pain Model Produced by MO Injection

The MO-induced pain model used in this study exhibited pain peaking over the first 30–40 min, i.e., primary hyperalgesia, consistent with previous reports (Ishikawa et al. 1999). This means that in the present model, continuous stimulation of pain receptors by neurogenic inflammation induced hyperalgesia in the acute and sub acute stages of tissue inflammation. The model used was, therefore, well suited to the analysis of molecular changes over time in pain response and was an excellent fit for the objectives of this study. The model shows appreciation for clinical applicability, exhibiting symptoms very similar to those associated with traumatic tissue injury in humans.

### Neuroglial Interactions and MAPKs Activity in the Initiation of Hyperalgesia

Numerous investigations have previously characterized the mechanisms underlying pathological pain, primarily by investigating pain-producing substances in peripheral sensory receptors and pain transmitters in the spinal cord, as well as the associated pain pathways. More recent attempts have gradually increased understanding through the investigation of neuroglial interactions and spinal sensory nerves and the modulation of spinal pain transmission and brain-derived inhibitors in relation to refractory hyperalgesia and allodynia. The research to date has suggested that following tissue damage, progression of pain receives contributions from neuroplastic changes in the processes of nociception, such as neuroglial interactions, in addition to peripheral sensory nerve inflammation, sympathetic nerve excitation, excessive transmission in spinal excitatory glutamate nerves, and morphological and physiological damage (dis-inhibition) to the inhibitory gamma amino butyric acid/glycinergic interneuron (Woolf and Salter 2000; Watkins et al. 2001; Svensson et al. 2003; Ji and Suter 2007; Zhou et al. 2008)

Woolf and Salter (2000) proposed that neuronal plasticity in spinal synaptic transmission is the primary mechanism behind persisting spontaneous pain and hyperalgesia following such peripheral tissue and nerve injury. The results of plentiful research conducted subsequent to this proposal suggest that MAPKs (ERK, p38-MAPK, JNK) (Svensson et al. 2003; Zhuo 2007) and BDNF derived from glial cell activation contribute to neuroplastic changes (Ji and Suter 2007; Merighi et al. 2008) A variety of intracellular signaling processes are modified during the progression of chronic pain. Zhuang et al. (2005) investigated the contributions of different glial cells to the time course of pain using pERK as a marker and proposed a profile based on their findings. Microglia associated with p38-MAPK increase and persist in a long-term manner following trauma (Tanga et al. 2004), partial sciatic nerve ligation (Zhuang et al. 2006), and spinal cord injury (Raghavendra et al. 2004).

This study revealed that microglial deactivators and the inhibition of p38-MAPK and NF-κB, which selectively activate inflammatory cells, produce analgesic effects for spontaneous pain occurring soon (1–4 h) after the onset of inflammation. This finding corresponds well with the analgesic effects that Svensson et al. (2003) achieved by administering p38-MAPK inhibitors to a rat model of formalin-induced inflammatory pain. Zhuang et al. (2005) also reported that analgesic activity was achieved with p38-MAPK inhibitor administration in a different model; a rat model of chronic pain by spinal nerve ligation. Our research integrates the contributions of transcription factor NF-κB activity and BDNF (discussed later) in such pain. More specifically, in the early stages of pain, the activation of inflammatory cell microglia in the spinal cord, the subsequent intracellular response, and the activation of signal p38-MAPK and NF-κB are related to increases in extracellular activators and the inflammatory cytokines prostaglandin E2, interleukin-1, interleukin-6, and tumor necrosis factor α (TNF-α) (Liu et al. 2007). Excessive cellular response, therefore, contributes to the early state of hyperalgesia.

Clinicians frequently encounter patients who develop hyperalgesia following the resolution of inflammation, spontaneous pain behavior, and other acute symptoms. This study revealed that inflammatory and spontaneous pain produced by MO paw injection results in thermal hyperalgesia at 24 h. JNK inhibition related to astrocyte markedly suppressed pain while the other drugs investigated failed to substantially suppress pain. The release of BDNF and S100β, an astrocyte-specific calcium binding peptide, can produce hyperalgesia by enhancing synaptic transmission in neighboring cells. Tanga et al. (2006) found that persisting increases in S100β in addition to glial fibrillary acidic protein contribute to pain following neuronal damage of the spinal cord in rats. Astrocytes also contribute substantially to pain sensitisation in the chronic state. Ridet et al. (1997) stated that spinal astrocyte expression is an important component of delayed and sustained hyperalgesia.

Hyperalgesia is, therefore, thought to occur when the elevated release of intervening transmission substances between neurons and glial cells (and particularly the elevated release of nitric oxide, ILs and other cytokines, and PGE2, which trigger microglial activation) increase the efficiency of neurotransmission. NF-κB is one of the most important due to its role as a transcription factor that controls the expression of many genes related to immune and inflammatory responses and promotes the gene transcription of many inflammatory mediators. Investigations in chronic pain have shown that NF-κB is activated in a variety of damaged neurons, including dorsal root ganglia following peripheral nerve injury and the spinal cord following its injury (Bethea et al. 1998; Ma and Bisby 1998). Such study has demonstrated the important role of the substance in neuroplasticity and pathological pain. Following onset of NF-κB activation, astrocytes are activated and BDNF, S100β, and other highly active substances modify synaptic transmission, leading to plastic changes in pain transduction networks.

### Contribution of BDNF to Progression of Hyperalgesia

BDNF is a protein essential for the differentiation, growth, and repair of neural networks following nerve injury and is a key modulator of functional expression in pain networks (Merighi et al. 2008). The present study showed that inhibitors of the BDNF receptor (Trk-B) suppress this in the early stage of pain facilitation, but do not play a role in delayed hyperalgesia. Previous investigation has shown that spinal administration of BDNF promotes excessive neurotransmission and closely contributes to neuroplasticity associated with pain (Cejas et al. 2000; Hayashida et al. 2008). Zhou et al. (2008) found that spinal BDNF administration to normal rats induced long-term potentiation of dorsal horn neurons and that this action was inhibited by p38-MAPK inhibitors. BDNF, therefore, plays a key role in the onset of hyperalgesia via the ERK, p38-MAPK, and NF-κB signaling pathways.

### Different Contribution of Glia-Related Signal Modulation Over Time in Hyperalgesia

The present study showed that glutamatergic activation associated with the increase in afferent nociceptive input immediately after tissue injury leads to the production of inflammatory mediators in the connected postsynaptic neurons. Furthermore, the nearby neuro-immune state in the neighborhood activates the microglia, thus leading to persistent pain. In this study, we found Iba-1 to be a reflection of microglia activation that occurred at the early stage, at 1–4 h after MO injection. This microglia activation is associated with p38-MAPK signaling and probably mediates initiation of hyperalgesia. Iba-1 is often used for demonstrating microglia activation and is also up-regulated by the ATP receptor P_2_X_4_ specifically, and blocking of this receptor results in decreased hyperalgesia. In addition, the underlying mechanism is thought to involve the stimulation of Trk-B located pre- and post-synaptically by BDNF produced in the early stage of neuroinflammation, with the resulting increased release of transmitters and elevated postsynaptic depolarization and nuclear signaling leading to greater BDNF production followed by activation of neighboring astrocytes. This is rooted in the efficacy of JNK-1 inhibitors in subacute pain, but not early stage of pain which is in good agreement with increase in GFAP (activated astrocyte) that was observed at 24 h. GFAP are well known as markers for astrocyte and appear to label most astrocytes in the spinal cord. The increased GFAP staining was attributed primarily to hypertrophy of astrocytes, rather than proliferation or migration. In addition, this delayed increase of GFAP may be related to neuro-astrocytic interactions during delayed chronic pain. Astrocyte activation promotes S100β and BDNF secretion, leading to delayed apoptosis and causing persisting hyperalgesia and even neuroplastic changes.

Svensson et al. (2003) showed a time-dependent contribution of microglial activation associated with p38-MAPK in the initiation of pathological pain. The effect of p38-MAPK inhibitors was examined in models of nociception and correlated with localization and expression levels of p38-MAPK in the spinal cord. A rapid increase in phosphorylated p38-MAPK in the spinal cord occurred following intrathecal administration of substance P or intradermal injection of formalin. Immunocytochemistry revealed that phosphorylated p38-MAPK-immunoreactive cells were predominantly present in lamina I–IV of the dorsal horn. Double-staining with markers for neurons, microglia, astrocytes, and oligodendrocytes revealed co-localization with microglia, but not with neurons or other glia.

This study revealed that in the model of persisting inflammatory pain, the release of p38-MAPK, NF-κB, and BDNF contributes to the initiation of hyperalgesia. This study also identified that JNK activation is subsequently developing thermal hyperalgesia. Our demonstration of the contribution of differences in signal modulation, related to different types of glia cells and leading to progression of chronic pain, represents an applicable finding for the clinical treatment of hyperalgesia.

## Conclusions

Persisting inflammatory pain is thought to cause derangements of neuroglia interaction in pain pathways and is followed by neuroplasticity, leading to chronic pain. This study revealed that microglia activation and its associated activation of p38-MAPK, NF-κB, and BDNF secretion contribute to initiation of hyperalgesia. We also showed that astrocyte activation accompanying JNK activation contributes to delayed hyperalgesia. Our demonstration of the usefulness of anti-inflammatory agents in the early stages and inhibitors of astrocyte-derived activating agents later in this progression of acute pain to chronic pain represents an important finding for the treatment of pain in each stage.
